# Mortality rate in rheumatoid arthritis-related interstitial lung disease: the role of radiographic patterns

**DOI:** 10.1186/s12890-021-01569-5

**Published:** 2021-06-30

**Authors:** Maria A. Nieto, Maria J. Rodriguez-Nieto, Olga Sanchez-Pernaute, Fredeswinda Romero-Bueno, Leticia Leon, Cristina Vadillo, Dalifer D. Freites-Nuñez, Juan A. Jover, Jose L. Álvarez-Sala, Lydia Abasolo

**Affiliations:** 1 Pneumology Department Hospital Clínico San Carlos, Madrid, Spain; 2 Universidad Complutense, Madrid, Spain; 3 Pneumology Department Hospital Fundación Jiménez Díaz UH, Madrid, Spain; 4 Centro de Investigación Biomédica en Red de Enfermedades Respiratorias (CIBERES), Instituto de Salud Carlos III, Madrid, Spain; 5 Rheumatology Department Hospital Fundación Jiménez Díaz UH, Madrid, Spain; 6 Instituto de Investigacion Sanitaria San Carlos (IdISSC) Hospital Clínico San Carlos, Calle Martin Lagos s/n, 4th, 28040 Madrid, Spain; 7 Rheumatology Department Hospital Clínico San Carlos, Madrid, Spain

**Keywords:** Rheumatoid arthritis, Interstitial lung disease, Mortality

## Abstract

**Background:**

To assess mortality rate (MR) and standardized mortality rate (SMR) of rheumatoid arthritis-related interstitial lung disease (RA-ILD) patients and to evaluate the role of radiographic patterns in mortality.

**Methods:**

A longitudinal multicentric study was conducted in RA-ILD patients from 2005 to 2015 and followed-up until October 2018 in Madrid. Patients were included in the Neumologia-Reumatología y Enfermedades Autoinmunes Registry, from diagnosis of ILD. The main outcome was all-cause mortality. The radiographic pattern at baseline [usual interstitial pneumonia (UIP), nonspecific interstitial pneumonia (NSIP), or others] was the independent variable. Covariables included sociodemographic and clinical data. Survival techniques were used to estimate MR, expressed per 1000 persons-year with their 95% confidence intervals [CI]. Cox multiple regression model was run to examine the influence of radiographic patterns on survival. SMR [CI] was calculated comparing MR obtained with MR expected in the general population of Madrid by indirect age-gender standardization.

**Results:**

47 patients were included with a follow-up 242 patients-year. There were 16 (34%) deaths, and most frequent causes were acute ILD exacerbation and pneumonia. MR was 64.3 [39.4–104.9], and 50% of the patients died at 8.3 years from ILD diagnosis. After adjusting for confounders, (UIP compared to NSIP was associated with higher mortality risk. The overall SMR was 2.57 [1.4–4.17]. Women of 60–75 years of age were the group with the highest SMR.

**Conclusions:**

RA-ILD is associated with an excess of mortality compared to general population. Our results support that UIP increases the risk of mortality in RA-ILD, regardless other factors.

## Background

Rheumatoid arthritis (RA) is a chronic autoimmune disease, characterized by persistent synovitis and systemic inflammation. The prevalence varies from 0.5 to 1% [1, 2], RA is associated with severe morbidity, impaired functional capacity leading to decreased quality of life [3] and mortality [4, 5].

Lung involvement can be observed as an extra-articular manifestation of the disease [6, 7], being interstitial lung disease (ILD) the most severe and prevalent pulmonary manifestation [8].

Rheumatoid arthritis-related interstitial lung disease (RA-ILD) may appear as a consequence of chronic immune activation promoting aberrant fibroproliferation, although it can also be precipitated by drugs or infectious agents [8]. As in the context of other interstitial pneumonias, RA-ILD can be categorized into injury patterns according to the different degrees of inflammation and fibrosis either in the radiological or in the histopathological assessment. Usual interstitial pneumonia (UIP) and non-specific interstitial pneumonia (NSIP) are the two most frequent radiological patterns observed in RA-ILD. ILD in RA is associated with significant morbidity, high healthcare resources utilization, and account for approximately 20% of all RA-associated mortality [9–12].

Over the past decade, it seems that mortality in RA has declined [5, 13–15]. New treatment strategies for RA with early and effective intervention leading to reduced disease activity [16] are probably the main cause of improvement. However, mortality is still increased in RA compared to general population [5, 13], being cardiovascular disease and ILD the mayor contributors of death in RA patients [17].

There are several observational studies assessing the mortality rate in RA-ILD patients, with a wide range of results [11, 12, 18–22]. Disparity may depend on heterogeneity in patient selection and characterization, differences in methods or length of reporting or even time-calendar, with possible changes in management strategies over study periods. To assess prognosis, it is also relevant to know which factors might affect survival. In this respect, the UIP radiographic pattern compared to NSIP has been identified as predictor of poor outcome in many RA-ILD studies [19, 23–26]. In contrast, no association between RA-ILD radiographic pattern and mortality was observed in a recent study [27]. A meta-analysis addressing this issue has been published, supporting that an UIP pattern seems to be associated with a higher mortality risk compared to other patterns [28]. However, it shows substantial heterogeneity with a variable methodological quality between the studies included. [19, 27]. Moreover, different criteria were used, both for the diagnosis of ILD and for the characterization of radiological patterns. Of note, those studies in which mortality could be adjusted for pulmonary function tests, did not find a difference in mortality between the two ILD patterns [19, 27, 29].

In order to provide new insight into the course of ILD in the context of autoimmune disorders in clinical practice, we have launched an observational multicentric registry (NEumología-REumatología y enfermedades Autoinmunes-acronym: NEREA-) in 2016 with participation of several Hospitals from Madrid. Briefly, NEREA gathers data from patients with rheumatologic autoimmune diseases with ILD, retrospectively collected from ILD diagnosis and updated during routine visits). With the current study we aim to better define the mortality rate and the standardized mortality rate (SMR) of RA-ILD patients from two tertiary centers included in the NEREA registry, using a strict and reproducible definition for the classification of ILD. In addition, we want to assess the role of radiographic patterns in the risk of mortality adjusting for confounders.

## Methods

### Setting

Two tertiary hospitals of the National Health System of the Community of Madrid, Spain, Hospital Clínico San Carlos (HCSC) and Hospital Fundación Jiménez Díaz (FJD) were the setting of the study, covering catchments area of approximately 400,000 people each. Rheumatology and Pneumology Services of each Hospital provide care to this entire population.

### Study design

This is a longitudinal multicentric observational study. Patients were included from Jan 2005-Oct 2015 and followed up until loss of follow-up, death, or end of study (October 2018).

### Patients

All patients were included from diagnosis of ILD and were included and followed up at special multidisciplinary RAD-ILD-units (Rheumatologic-Autoimmune-Disease-Interstitial Lung Disease) carried out by a pneumologist and a rheumatologist. These consultations were included in the usual clinical practice for the management of these patients. They were followed according to clinical needs, but usually every 3 or 6 months once the patient was considered stable. The evaluation was performed by respiratory function tests at baseline and approximately every 6–12 months, and by CT scans at baseline. CT scans could be repeated during the follow-up according to the medical criterion. Patient data were recorded in NEREA Registry (NEumologia-REumatología y Enfermedades Autoinmunes). Briefly, this register has Rheumatology Autoimmune Disease/-Interstitial Lung Disease-(RAD-ILD) data from patients. Every RAD was diagnosed according to EULAR and ACR classification criteria and an ILD was diagnosed according to the guides of the scientific societies European Respiratory Society/American Thoracic Society (ERS/ATS) [30]. NEREA collected information from routine clinical practice and do not involve additional consultations or tests. NEREA accumulate retrospective and prospective information from study patients [31].

Study inclusion criteria were patients from NEREA with diagnosis of rheumatoid arthritis (RA) according to ACR/EULAR 2010 RA classification criteria [32] and diagnosis of ILD [30]. For inclusion, the diagnosis of an ILD had to be confirmed by an expert pneumologist after performing a standardized systematic diagnostic workup in accordance with current guidelines [30]. Patient data in this project were obtained during routine clinical practice and informed consent to participate was signed. The study was conducted in accordance with the Declaration of Helsinki and Good Clinical Practices and was approved by the Hospital Clinico San Carlos institutional review board (internal number 17/272-E), and for the Hospital Universitario Fundación Jiménez Díaz IIS institutional review board (internal number CDC_EOH043).

### Variables

The primary outcome was all-cause mortality obtained from the Hospital information Systems in both Hospitals (HIS). When data was available, both, the cause and the date of death were recorded. The independent variable was RA-ILD radiographic patterns at baseline, according to high-resolution computed tomography images (HRCT) as follows: UIP, NSIP and others (cryptogenic organizing pneumonia (COP) and other idiopathic interstitial pneumonia (IIP).

The following covariates were included: (a) sociodemographic characteristics: (b) clinical characteristics: baseline comorbidities, duration of RA disease, body mass index (BMI), smoking habit, (c) disease related variables: rheumatoid factor (RF), anti-citrullinated antigens antibodies (ACPA) and erythrocyte sedimentation rate (ESR) at baseline, (d) pulmonary functional tests (PFT) at baseline and at every follow-up visit (% predicted forced vital capacity [FVC%], % predicted single-breath carbon monoxide diffusing capacity [DLCO%]; and (e) treatments prescribed prior to ILD diagnosis and during follow-up, including. (e1) glucocorticoids: (e2) disease modifying antirheumatic drugs (DMARDs) comprising conventional synthetic (cs) DMARDs (Azathioprine, Mycophenolate-mofetil, Cyclophosphamide, Methotrexate, Leflunomide, Antimalarials, Sulfasalazine) and biologic agents (bDMARDs: TNF inhibitors [Anti-TNF], Rituximab [RTX], Abatacept [ABA], Tocilizumab [TZL]); (e3) antifibrotic agents (Pirfenidone); and (e4) oxygen therapy (O2). Prior use of therapies was defined as: (e1) any use up to 2 months before ILD diagnosis, and (e2) any DMARD administered for at least 2 months from RA diagnosis and before ILD onset. Concurrent use of therapies was defined as: (e1); used a minimum of 2 months at any time from ILD diagnosis and; (e2) any DMARD used during the study period, and recorded as starting-to-ending dates. (f) Fibrosis development over time (determined by HRCT: yes/no); (g) calendar time: dividing the time of ILD diagnosis in 4 year intervals (from 1st. Jan 2005 until 31th. Dec 2008; 1st. Jan 2009 until 31th. Dec 2011; and 1st. Jan 2012 until Oct 2015).

### Statistical analysis

Patient´s characteristics were described as mean and standard deviation for continuous variables, while proportions were shown for categorical variables.

Survival techniques were used to estimate the incidence rate of deaths (MR). expressed per 1000 persons-year with a 95% confidence interval [CI]. The time of observation comprised the elapsed time between the RA-ILD first visit date and the date of patient's death, loss of follow up or end of study. Survival over time was evaluated using Kaplan–Meier curves and the log-rank test.

Cox bivariate analyses were done to assess differences between mortality risk and covariates. Cox multivariate regression model (adjusted for age, gender, disease severity, smoke and calendar time) were run in a stepwise manner to examine the possible influence of radiographic patterns on survival. Fibrosis development was assessed in a time-dependent manner. Results were expressed by hazard ratio (HR) and [CI]. Proportional hazard assumption was tested using Schoenfeld residuals and the scaled Schoenfeld residuals.

The standardized mortality rate (SMR) was estimated by indirect age and genderstandardization, dividing the observed MR by the expected ones. Expected cases were calculated from incident cases of death in the general population of the Madrid Community (Spain) for the year 2018.The 95% confidence intervals were estimated using the Poisson distribution.

A two-tailed *p* value under 0.05 was considered to indicate statistical significance.

## Results

Forty seven RA-ILD patients, 29 from HCSC and 18 from FJD, were included. The mean follow-up was 5.3 ± 2.7 years, with a total follow up of 242 patients-year and a maximum follow-up of 12 years. Table 1 includes a detailed description of the patients. Many of them were women in their seventies. In most patients (89.4%) RA diagnosis preceded ILD, being 11.9 ± 11.9 years the mean lag time.

**Table 1:** Sociodemographic and clinical characteristics of the patients

	All (n = 47)
Women n (%)	33 (70.2)
Age at ILD diagnosis mean ± SD, years	72.04 ± 8.6
BMI median [p25–p75]	27.1 [24.1–31.1]
Lag time to ILD diagnosis median [p25–p75], years	6.9 [1.1–17.3]
Smokers (active and formers); n (%)	25 (54.3)
Rheumatoid Factor + (n = 46); n (%)	43 (93.5)
Rheumatoid Factor median [p25–p75]	205 [76.7–354]
Anti-CCP + (n = 35); n (%)	29 (82.8)
Anti-CCP median [p25–p75]	400 [78–593]
ESR median [p25–p75]	42 [22.5–55]
Comorbidity; n (%):	
Hypertension	26 (55.3)
Cholesterol	20 (42.5)
CVD	11 (23.4) 4
Cancer	(8.5)
Diabetes mellitus	6 (12.8)
Congestive heart failure	4 (8.5)
Sleep apnea syndrome	3 (6.4)
Depression	4 (8.5)
Renal failure (dialysis)	1 (2.1)
Liver disease	4 (8.5)
Hypothyroidism	5 (10.6)
Emphysema	11 (23.4)
PTF parameters	
FVC% median [p25–p75]	91 [76–111]
DLCO% median [p25–p75] (n = 43)	65.8 [48.6–82.7]
Radiographic ILD pattern n (%)	
UIP	26 (55.3)
NSIP	16 (34)
Others	5 (10.7)
Therapy during the follow-up, n (%)	
Corticoids	45 (95.5)
O2	12 (25.5)
csDMARDs	
AZA/MMF	17 (36.2)
MTX	13 (27)
CyC	2 (4.2)
Other csDMARDs	23 (48.9)
bDMARDs	
Anti-TNF	12 (25%)
RTX	22 (46.8)
ABA	11 (23.4)
TZL	2 (4.2)
PIRF	2 (4.2)

*Centers* HCSC or Hospital Clínico San Carlos and FJD or Hospital Fundación Jiménez Díaz, *ILD* interstitial lung disease, *BMI* body mass index, *ESR* eritrocite sedimentation rate, *FVC%* predicted forced vital capacity, *DLCO%* predicted diffusing capacity of the lungs for carbon monoxide, *UIP* usual interstitial pneumonia, *NSIP* nonspecific interstitial pneumonia, *CVD* cardiovascular and cerebrovascular disease (ischemic heart disease, peripheral vascular disease and cerebrovascular disease), *csDMARDs* conventional synthetic disease modifying antirheumatic drugs, *CYC* cyclophosphamide, *MTX* methotrexate, *AZA* azathioprine, *MMF* Micophenolate mofetil, *Other csDMARDs* Leflunomide; Antimalarials; Sulfasalazine; *bDMARDs* biologic DMARDs, *Anti-TNF* TNF inhibitors including ADA (Adalimumab), *ETN* Etanercept, *IFX* Infliximab, *CERTO* Certolizumab, *ABA* Abatacept, *RTX* Rituximab, *TZL* Tocilizumab, *PIRF* the antifibrotic agent Pirfenidone; *O*_*2*_ Oxygen

The mean baseline FVC% was 94.3 ± 19.9, with 2 patients showing FVC% values under 70%. In contrast, DLCO%, baseline levels were between normal values (> 80%) in 28% of the patients, while 35% had a mild DLCO% deterioration (60–80%), another 28% had moderate reduction (40–60%) and the remaining 9% showed severe DLCO% deterioration (< 40%) [33]. Regarding ILD radiological patterns, UIP was the most frequent, followed by NSIP. The number of patients taking glucocorticoids increased from 72% before ILD diagnosis to 91.5% during follow-up. None of the patients were using supplemental oxygen at study entry, whereas 25% needed it during follow-up. With respect to the use of DMARDs before ILD diagnosis, 74% of the patients were on Methotrexate, 25% were taking Leflunomide, 23% Antimalarials and 21% Azathioprine. For biologic agents, 30% were taking Anti-TNF, none was on RTX and 2% were being treated with ABA. From ILD onset and during follow-up (Table 1) there was an increase in the use of RTX, Azathiprine and ABA. On the other hand, there was a decrease in the use of Methotrexate and Anti-TNF.

Death occurred in 16 patients (34%) during the whole follow-up. The most frequent cause was progression of ILD, acute exacerbation of ILD or pneumonia (n = 10; 62.5% of the deaths), followed by abdominal sepsis (n = 2; 12.5%), neoplasia (n = 1; 6.25%), cardiological disease (n = 1; 6.25%), cognitive deterioration (n = 1; 6.25%) and no specific cause in an institutionalized elderly (n = 1; 6.25%).

**Fig. 1:**
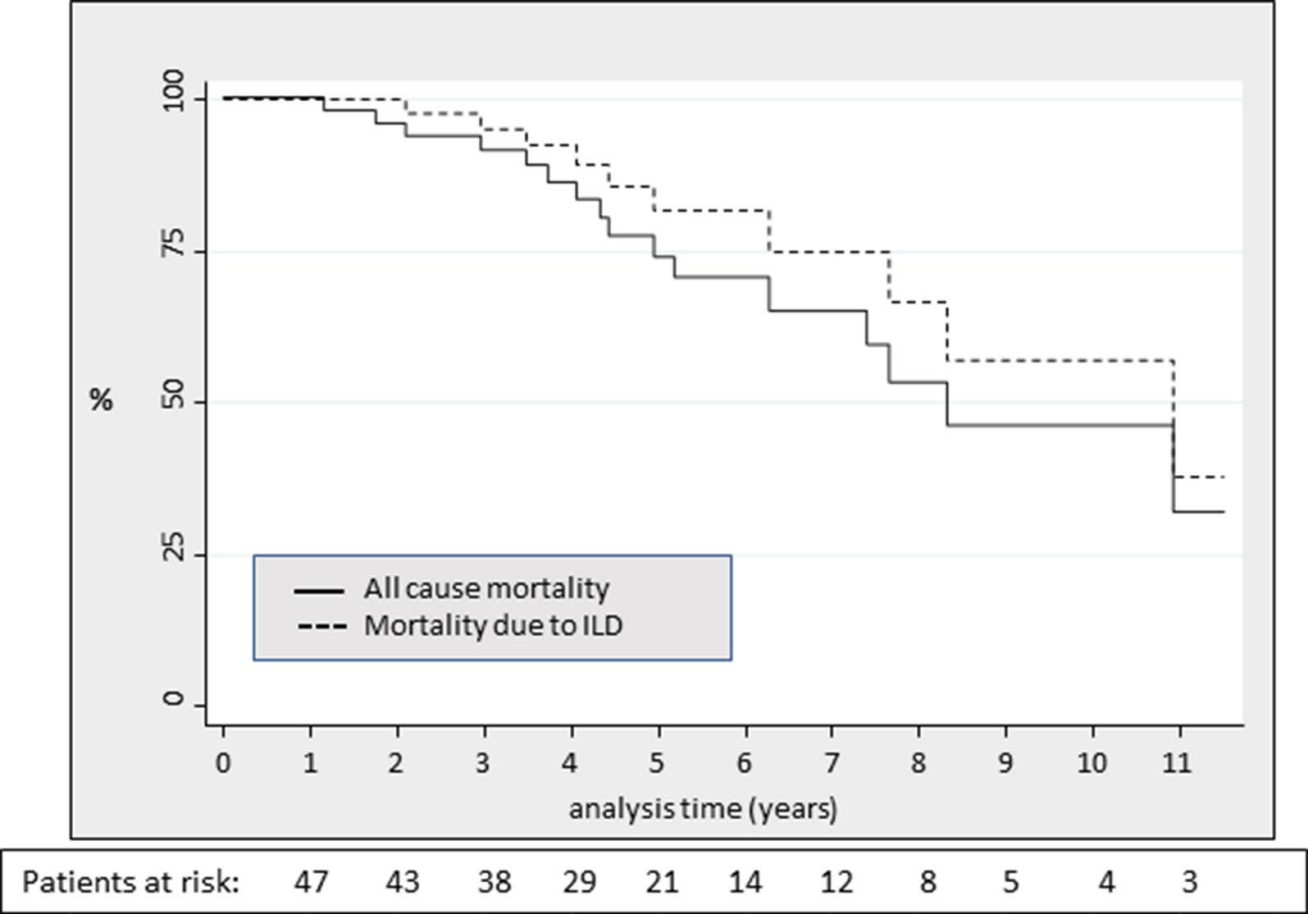
Kaplan–Meier survival estimate curve in RA-ILD patients

The MR estimated in all-cause mortality was 64.3 [39.4–104.9] per 1000 patients-year. As shown in the Kaplan Meier survival curve (Fig. 1), patients were free of deaths until 1.5 years after diagnosis of ILD; there was a 10% of mortality at 3 years, a 30% at 6 years, and a 50% at 8.3 years from ILD diagnosis. Considering the deaths directly attributable to ILD, the specific MR was 41.4 [22.3–76.9], with a median survival time of 10.9 years.

**Fig. 2:**
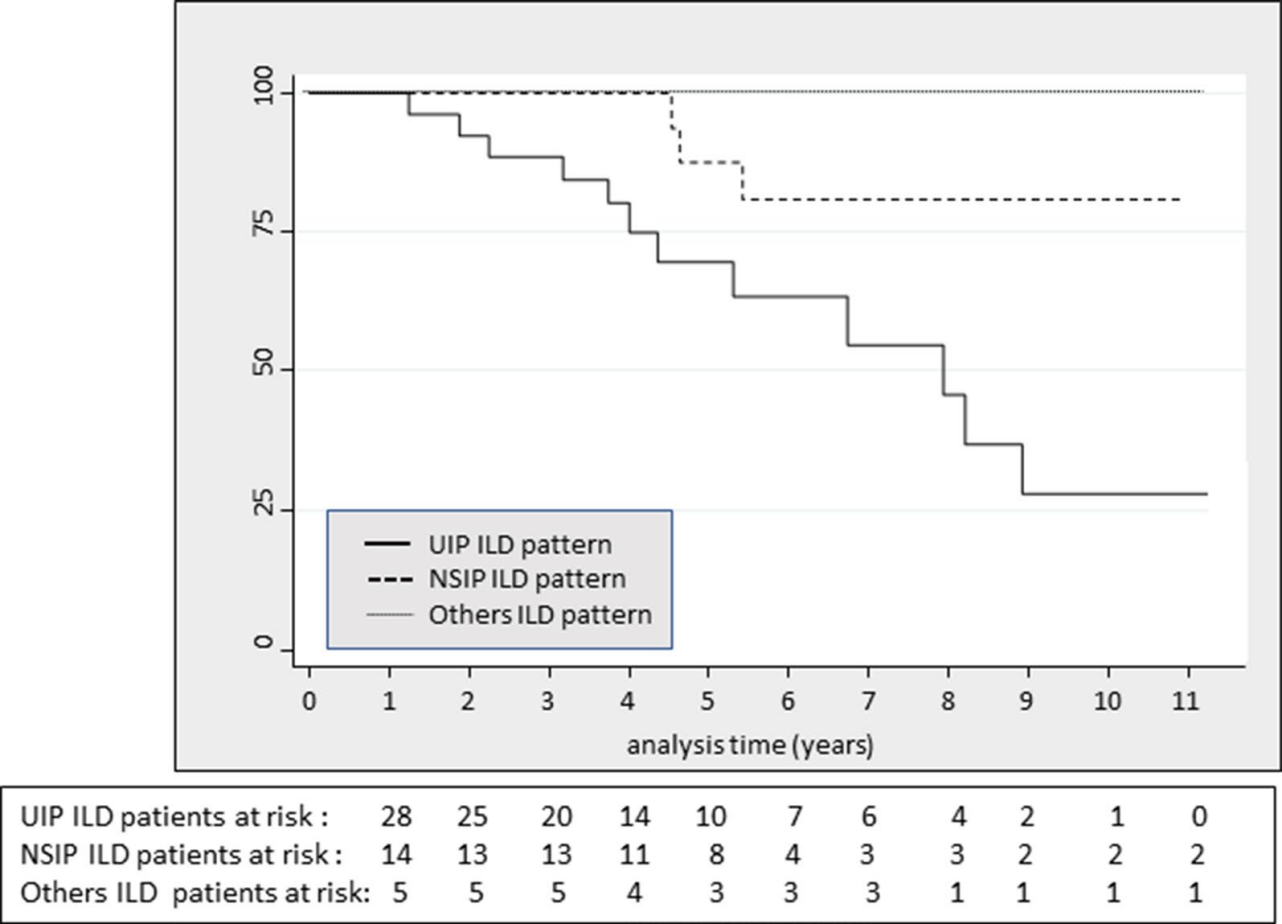
Kaplan–Meier survival estimate curve by radiographic ILD radiological pattern

Table 2 shows the MR in all cause-mortality by different variables. Of interest, all deaths occurred in RF positive cases, being MR slightly higher for RF titers over 300 IU/ml (95.7 [43–213.2]) versus lower titers (50.3 [26.2–96.7]). Conversely, we did not observe differences in MR between high and low Anti-CCP titers (> 300: 50.2 [18.8–133]; < 300: 52.1 [21.7–125]), although it was higher in negative Anti-CCP cases (MR: 91.1 [29.3–282]) compared to positive (MR: 42.1 [18.9–93.6]). Regarding to DLCO%, those patients with higher levels showed lower crude MR, as expected. Finally, concerning radiological patterns, MR was higher in patients with UIP (Fig. 2).

**Table 2:** Incidence rate of all-cause mortality by different variables in RA-ILD patients

	Patient-year	Events	MR	95 CI
All cause mortality	249	16	64.3	39.4–104.9
Sex				
Women	189	10	52.8	28.4–98.2
Men	60	6	100.7	45.2–224.2
Age at diagnosis, years:				
< =60	27.4	0	–	–
61–74	120	5	41.6	17.3–100
> =75	101	11	108.6	60.1–195.6
Center				
FJD	94	7	74.5	35.5–156.4
HCSC	155	9	58.1	30.2–11.6
Positive rheumatoid factor				
No	22	0	–	–
Yes	227	16	67.0	40.4–11.2
Smokers				
Never	115	7	61.0	29.1–128.0
Active and formers	126	9	71.3	37–137
Missing data	8	0	–	–
PTF parameters:				
DLCO%:				
< 60	91.5	10	109.3	58.5–2.3.2
> 60	154.5	6	38.8	17.4–86.4
Missing data	3	0	–	–
Radiographic ILD pattern				
UIP	131	13	98.6	57.3–169.9
NSIP	83	3	36.4	11.7–112.8
Others	35	0	–	–

*Centers* HCSC or Hospital Clínico San Carlos and FJD or Hospital Fundación Jiménez Díaz, *ILD* Interstitial lung disease, *UIP* usual interstitial pneumonia, *NSIP* nonspecific interstitial pneumonia

Bivariate and multivariate analyses are detailed in Table 3. After adjusting for confounders, UIP pattern was associated to a higher risk of mortality compared to NSIP pattern. Men compared to women lost statistical significance (HR: 073; p = 0.5) in the multivariate analysis. Fibrosis development and basal active disease dropped from the final model (p = 0.6). Proportionality of these regression models was tested with a *p* value = 0.9.

**Table 3:** Influence of radiographic patterns and other variables on survival in RA-ILD patients: bivariate and multivariate analysis

	HR	95 [CI]	p	HR	95 [CI]	p
Sex: men versus women	2.83	1.02–7.8	0.046	0.73	0.25–2.12	0.5
Age, years	1.08	1.01–1.16	0.019	1.16	1.03–1.3	0.013
Center: HCSC versus FJD	0.65	0.24–1.78	0.4	–	–	–
Smokers: active/formers versus never	1.34	0.5–3.5	0.5	5.6	1.12–28.7	0.03
PTF parameter: DLCO% > 60	0.38	0.14–1.05	0.06	0.11	0.03–0.44	0.002
Fibrosis development (yes)	6.8	0.94–50.1	0.058	–	–	–
Basal disease activity (ESR > 41)	2.1	0.7–6.5	0.2	–	–	–
Calendar time:						
2005–2008	1	–	–	1	–	–
2009–2011	1.9	0.56–5.65	0.2	3.8	1.14–13.2	0.03
2012–2015	0.25	0.02–2.18	0.2	0.15	0.01–1.89	0.14
Radiographic ILD pattern: NSIP and Others) versus UIP	0.15	0.03–0.68	0.014	0.16	0.04–0.63	0.008

*Centers* HCSC or Hospital Clínico San Carlos and FJD or Hospital Fundación Jiménez Díaz, *ILD* Interstitial lung disease, *DLCO%* predicted diffusing capacity of the lungs for carbon monoxide, *UIP* usual interstitial pneumonia, *NSIP* nonspecific interstitial pneumonia

The overall SMR was 2.57, resulting in an excess of mortality in our RA-ILD patients compared to general population. In Table 4, we observe that men and women as well as those patients between the ages of 75 and 84 years had an excess of mortality, while this effect disappeared over 85 years. After stratifying by age and gender, women from 65 to 74 years and men from 75 to 84 years had an excess of mortality. Specifically, women between 60 and 75 years were the group with the highest SMR.

**Table 4:** Comparations of mortality between the AR-ILD from the NEREA Registry and the Madrid Community general population using standardized mortality ratios (SMR) based on sex and age

		E	SMR	95 CI
All cause	16	6.23	2.57	1.4–4.17
Sex				
Men	6	1.95	3.08	1.13–6.7
Women	10	3.61	2.77	1.3–5.09
Age at 2018 or end of study, years:				
55–64	0	0.011	0	–
65–74	3	0.74	4.05	0.83–11.84
75–84	8	2.94	2.71	1.17–5.35
> 85	5	4.72	1.05	0.34–2.4
Age at 2018 or end of study in women, years:				
55–64	0	0.06	0	–
65–74	3	0.44	6.8	1.4–19.8
75–84	3	1.31	2.3	0.5–6.7
> 85	4	3.33	1.2	0.3–3.1
Age at 2018 or end of study in men, years:				
55–64	0	0	–	–
65–74	0	0.16	0	–
75–84	5	1.6	3.1	1.01–7.3
> 85	1	1.04	0.96	0.02–5.3

E: cases expected in general population. O: cases observed in RA-ILD patients of the study

*O* observed, *E* expected, *SMR* standardized mortality rate

## Discussion

In this real-world longitudinal study from 2005 to 2018, all-cause mortality among patients with RA-ILD was significantly higher than in general population of Madrid Community matched by gender and age. Our results seem to indicate the role of radiological patterns in mortality risk regardless other factors.

Patients from our study might be considered representative of the RA-ILD population. In consonance with previous reports, they were in their late sixties and most of them were RF and/or Anti-CCP positive [11, 18, 34–36]. UIP was the most prevalent radiological pattern, followed by NSIP [19, 24, 37].

It has been published that ILD results in a decreased lung function and is a leading cause of death among RA patients, after cardiovascular disease [38]. Within RA-ILD patients from NEREA registry, the most frequent cause of death was respiratory failure [12].

We confirm that RA-ILD is a life-shortening condition. In our study 10% and 30% of RA-ILD patients had died by 3 and 6 years respectively after diagnosis of ILD, with a median survival of 8.2 years. Similar findings were shown in the studies from Raimundo et al. [36] and Solomon et al. [19]. This data appears to be somewhat better than those reported in many previous studies [11, 21, 36]. Several of them are dated at the pre-biologic era. It could be considered that newer DMARDs therapies for RA decrease the severity of these patients including the extra-articular manifestations. Alternatively, the relative improvement could be related to the development of diagnostic RA-ILD criteria, but also to an increased disease awareness of this complication, leading to an active screening and an earlier detection.

Concerning risk factors of mortality, older age is the most consistent variable identified as a significant predictor of a poor prognosis [10, 18, 19, 21, 27, 39, 40]. Other variables associated to mortality in RA-ILD include male gender, ILD disease severity, autoantibody profiles [41], RA disease activity and a UIP pattern [19, 23–37]. Regarding the latest, Sight points out in a meta-analysis [28], several factors that may confound analyses of RA-ILD pattern and mortality, underlining the need of including all of them. In this sense, we included age, sex, disease activity, calendar time at ILD diagnosis and smoking habit, along with the radiographic pattern. In our study all dead patients were RF positive, thus RF was not included in the model. Our results corroborate the relevance of age and smoking habit, and have to highlight the importance of the pulmonary function test [28, 39], and the relevance of the radiological pattern when considering prognosis of RA-ILD.

Interestingly, many authors have studied the relationship of radiological disease extension with death risk [24, 42–44]. We also included extension of disease in terms of fibrosis development in the multiple regression analysis, but it drooped from the final model. This variable was measured as the appearance of fibrosis over time, regardless the extension. Of note, in clinical practice there is currently no standardized quantitative method providing an objective and reproducible measurement of radiological extension and/or an accurate assessment of the amount of fibrotic changes [28, 39]. Nevertheless, the semiquantitative CT score proposed by Goh et al. [45] and validated in RA-ILD by Ito Yuhei et al. [46] could be a valid alternative in clinical practice for prospective studies.

All published evidence agrees that ILD shortens survival in RA patients [11, 12, 19, 35, 36]. In this study, the MR was estimated in 64.3 per 1000 patient’s year, being almost three times superior to MR reported in other studies of RA population without ILD [5, 11, 47]. Going further, our study shows the excess of mortality compared to general population in the 74- to 85-years of age group [12]. Specifically, by gender, women from 60 to 75 years were the group with the highest SMR.

The results of our study should be interpreted considering several limitations. The retrospective nature of the design is a major limitation of our study. Another limitation is sample size, since the prevalence of clinically significant RA-ILD can rise to 10% of RA patients [12]. In this sense, our findings could be corroborated in further studies with larger sample sizes. As strengths, we include non-selected well characterized AR-ILD patients, using a classification criterion for ILD with clinical and sociodemographic details at onset, reflecting clinical practice from two hospitals, with a long-term follow up. We can conclude that RA-ILD is associated with a higher MR than general population. Taking account of the results of the ongoing study, the underlying pattern of ILD seems to be important for its prognostic value. Thus a better fibrosis control, good maintenance of respiratory function, as well as avoiding tobacco consumption, could help these patients to improve their survival.

## Conclusions

Rheumatoid arthritis-related interstitial lung disease (RA-ILD) is associated with decreased survival. In this real-world longitudinal study mortality among patients with RA-ILD was significantly higher than in general population of Madrid. Our results support that usual interstitial pneumonia pattern increases the risk of mortality regardless other factors.

## Data Availability

The datasets used and/or analysed during the current study are available from the corresponding author on reasonable request.

## Abbreviations

MR: Mortality rate
SMR: Standardized mortality rate
RA-ILD: Rheumatoid arthritis-related interstitial lung disease
NEREA: Neumologia-Reumatología y Enfermedades Autoinmunes Registry
UIP: Usual interstitial pneumonia
NSIP: Non-specific interstitial pneumonia
CI: Confidence interval
RA: Rheumatoid arthritis
HCSC: Hospital Clínico San Carlos
FJD: Hospital Fundación Jiménez Díaz
RAD-ILD-units: Rheumatologic-Autoimmune-Disease-Interstitial Lung Disease
ERS/ATS: European Respiratory Society/American Thoracic Society
HRCT: High-resolution computed tomography images
COP: Cryptogenic organizing pneumonia
IIP: Idiopathic interstitial pneumonia
BMI: Body mass index
RF: Rheumatoid factor
ACPA: Anti-citrullinated antigens antibodies
ESR: Erythrocyte sedimentation rate
PFT: Pulmonary functional tests
FVC: Predicted forced vital capacity
DLCO: Single-breath carbon monoxide diffusing capacity
DMARDs: Disease modifying antirheumatic drugs
Anti-TNF: TNF inhibitors
RTX: Rituximab
ABA: Abatacept
TZL: Tocilizumab
O_2_: Oxygen therapy
